# Supplementary figures and images for: Moderating the link between discrimination and adverse mental health outcomes: Examining the protective effects of cognitive flexibility and emotion regulation
Source: PLoS One. 2023 Oct 10;18(10):e0282220. doi: 10.1371/journal.pone.0282220 (PMC10564165; doi:10.1371/journal.pone.0282220)

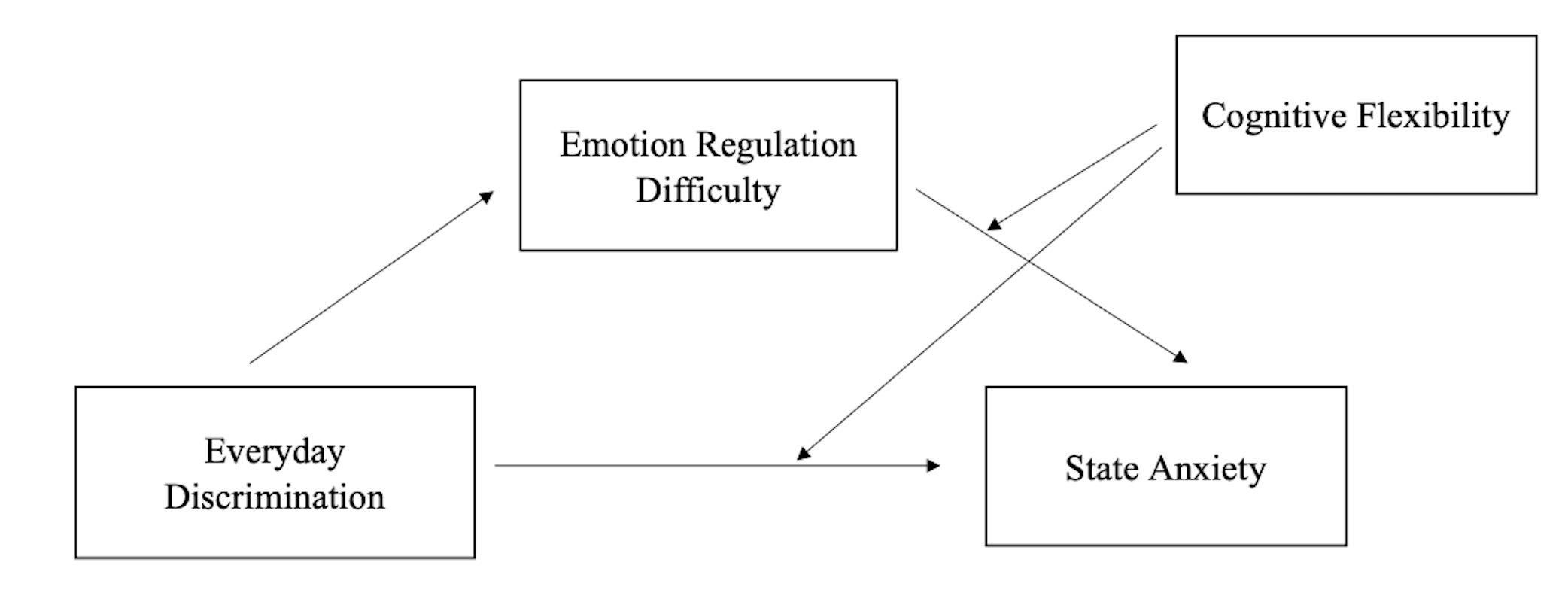

Supplement: S1 Fig — (TIF) [file pone.0282220.s001.tif]

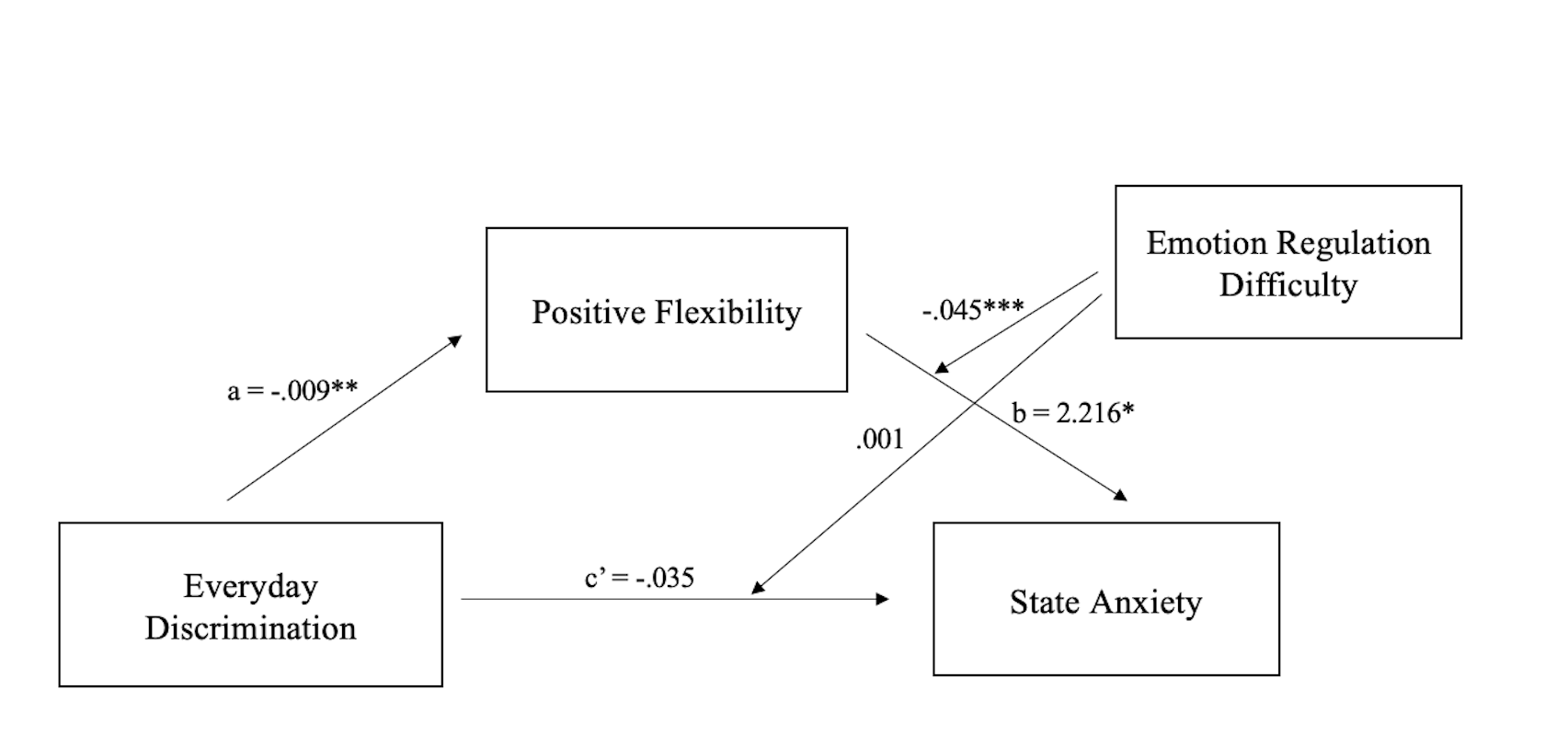

Supplement: S2 Fig — *p < .05, **p < .01, ***p < .001. (TIF) [file pone.0282220.s002.tif]

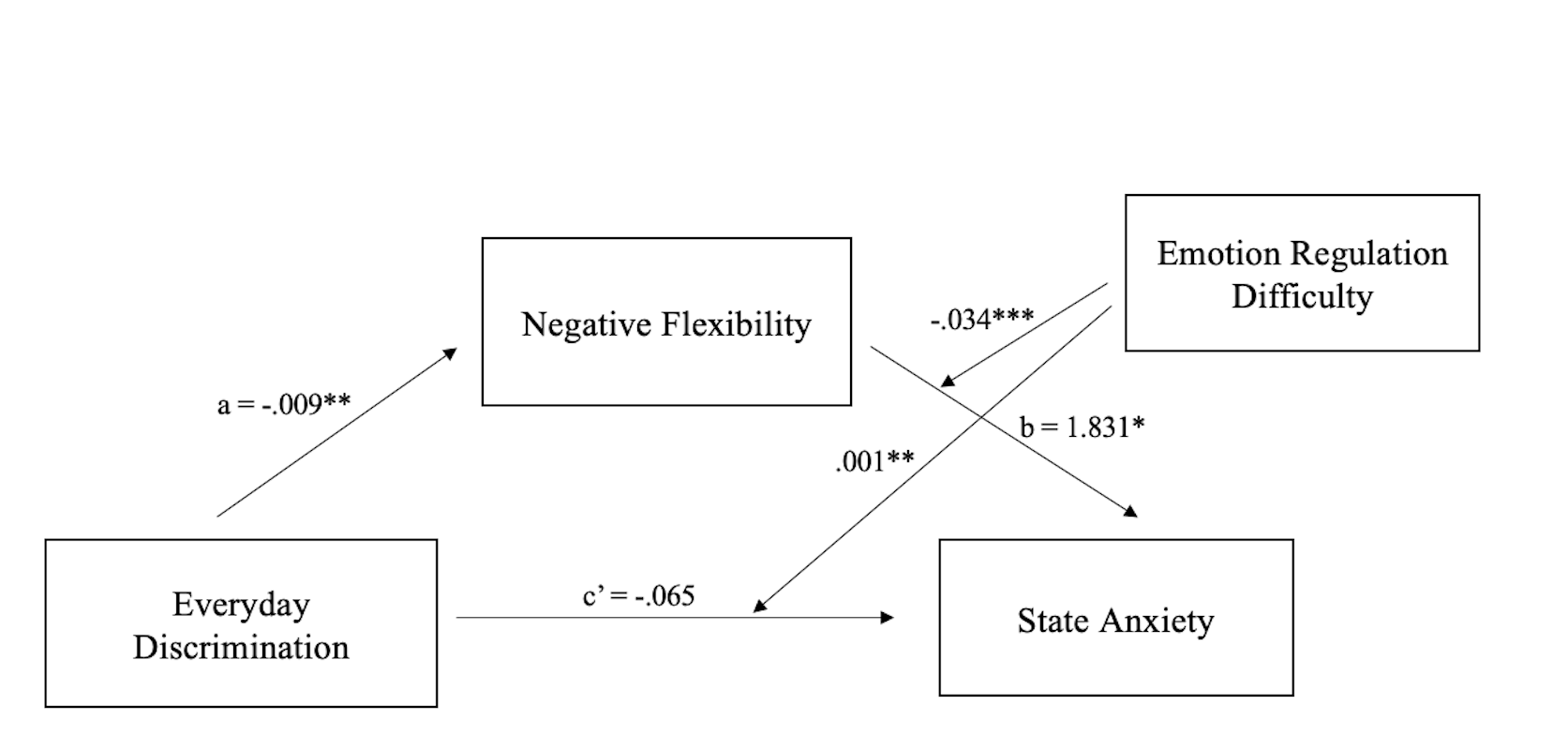

Supplement: S3 Fig — *p < .05, **p < .01, ***p < .001. (TIF) [file pone.0282220.s003.tif]
